# Effect of Dietary Cholesterol, Phytosterol, and Docosahexaenoic Acid on Astaxanthin Absorption and Retention in Rainbow Trout

**DOI:** 10.1155/2024/8265746

**Published:** 2024-10-15

**Authors:** Yang Jin, Keshuai Li, Jon Olav Vik, Marie Hillestad, Rolf Erik Olsen

**Affiliations:** ^1^Department of Animal and Aquacultural Sciences, Norwegian University of Life Sciences, Aas, Norway; ^2^BioMar AS, Trondheim, Norway; ^3^Faculty of Chemistry, Biotechnology and Food Science, Norwegian University of Life Sciences, Aas, Norway; ^4^Department of Biology, Norwegian University of Science and Technology, Trondheim, Norway

**Keywords:** astaxanthin, cholesterol, docosahexaenoic acid, phytosterol, proteome, transcriptome

## Abstract

Astaxanthin (Ax) determines the flesh redness of a salmonid fish which is the most desirable quality indicator by consumers. Fish cannot synthesize Ax de novo, therefore, the only way to increase flesh redness is to increase dietary input or improve the absorption and retention rate of dietary Ax. As a hydrophobic carotenoid, the absorption of Ax can be modulated by other lipid molecules in the diet. The present study explored the effect of three lipids, cholesterol (CH), phytosterol (PS), and docosahexaenoic acid (DHA) on Ax absorption, transport, and retention in rainbow trout. Dietary CH significantly improved Ax absorption by elevating plasma Ax levels (*p* < 0.05); however, it had no effect on the whole body Ax or flesh color. Dietary PS appears to inhibit Ax absorption since fish had significantly (*p* < 0.05) reduced whole body Ax. Dietary DHA appeared to have no effect on Ax absorption or retention. By comparing intestinal transcriptomes, a low density lipoprotein receptor (*ldlr*) gene was significantly downregulated in fish fed the CH diet as compared to the control diet. Since LDLR protein plays a major role in plasma lipoprotein turnover, we hypothesized that the inhibition of *ldlr* gene by high dietary CH resulted in higher retention of plasma Ax. The elevation of plasma Ax was not reflected in higher flesh coloration, which suggested other limiting factors governing Ax retention in the muscle. On the other hand, the transcriptomic and proteomic analyses found no changes of genes or proteins involved in Ax absorption, transport, or excretion in fish fed PS or DHA diets as compared to the control diet. In conclusion, this study has suggested that CH promotes Ax absorption by regulating lipoprotein retention and provide evidence for improving Ax absorption via dietary modulation.

## 1. Introduction

Astaxanthin (Ax) is an important feed ingredient for commercial salmon aquaculture due to its role in determining the red flesh color of the fish and its effectiveness as an antioxidant. The dietary inclusion of Ax is highly correlated with flesh redness in salmon. However, the retention of Ax in muscle is generally low, with retention rates ranged from 3% to 18% in rainbow trout [1–3] and 3.9% to 12% in Atlantic salmon [3, 4]. Considering that the pink-red flesh color is the most desirable quality indicator among customers [5], it has become necessary for the salmon farmers to add high levels of dietary Ax to achieve this color.

As a hydrophobic xanthophyll, Ax is absorbed and transported similarly to other dietary lipids in the gastrointestinal tract. Ingested Ax is mostly located in the core of lipid emulsions together with other nonpolar lipids such as triacylglycerols (TG) and cholesterol (CH) [6, 7]. The Ax is then transferred to bile salt micelles and absorbed by intestinal enterocytes. The uptake of Ax involves several proteins such as Scavenger receptor class B Type 1 (SR-B1) [8, 9], cluster determinant type 36 (CD36) [10, 11], and Niemann Pick C1 Like 1 (NPC1L1) [9], all of which are also essential for CH absorption [12]. Following absorption, Ax is packed into lipoproteins and secreted to the circulation where they are taken up and stored in peripheral tissues such as muscle. Unlike other white fish living in the same habitat such as Atlantic cod, salmonids absorb and deposit large amounts of Ax in muscle cells, resulting in their distinct color [4].

The intestine is suggested to be the key tissue which limits the retention of Ax in salmonids [11, 13]. Enzymatic cleavage by *β*-carotene 15,15′-oxygenase (BCO1) is the main degradation pathways for carotenoids in intestinal enterocytes [14, 15]. The BCO1 protein has also been suggested to have the similar cleavage activity on Ax, which limits the release of Ax from enterocytes into circulation and its retention in the muscle tissue [13]. Another membrane proteins adenosine triphosphate (ATP)-binding cassette sub-family G member 2 (ABCG2) has also been found to be associated with the flesh color of salmonids [16]. Given that ABCG2 in mammals is located on the apical side of the intestinal lumen and acts as a xenobiotic efflux pump [17], it was hypothesized that ABCG2 may excrete absorbed Ax back to the intestinal lumen of the salmonids gut [16].

Due to its hydrophobic properties, it is not surprising that the absorption of Ax can be modulated by other dietary lipids. CH and phytosterol (PS) are structurally similar to Ax, therefore it is possible that PS and CH could compete with Ax for absorption through the NPC1L1 pathway and for excretion through ABC-transporters. Furthermore, omega-3 fatty acids such as docosahexaenoic acid (DHA; 22:6*n*-3) and eicosapentaenoic acid (EPA; 20:5*n*-3) are potential NPC1L1 inhibitors and could reduce Ax absorption and transport [18]. The present study tested the hypothesis that dietary inclusion of CH, PS, and DHA affect Ax retention in rainbow trout. Fish were fed diets supplemented with either CH, PS, or DHA and their flesh color and Ax levels were quantified. Transcriptome and proteome of the fish intestine were also measured to understand the molecular mechanisms underlying the beneficial or inhibitory effect for Ax absorption and retention in response to the three diets.

## 2. Material and Methods

### 2.1. Fish Husbandry and Sampling

All procedures were conducted in accordance with the Danish Animal Experiment statutory declaration LBK nr 1306 of 23/11/2007 (in Danish: LBK nr 1306 af 23/11/2007). The feeding trial with rainbow trout in brackish water (17.6 ppt) was performed in 1 m^3^ tanks at Aquaculture Technology Center (ATC) Hirtshals, Denmark, for a total of 59 days from August 26^th^ to October 29^th^, 2020. The fish were separated into 12 tanks (three replicate tanks per dietary group) with 40 fish per tank. The fish had an initial weight of 300 g and were acclimated to 14–15°C in seawater for 14 days before the feeding trial started. The fish were fed at 1% of body weight per day. The fish were maintained in water at 14.6°C, pH 7.2, and 100% oxygen saturation on a 12:12 h light:dark cycle. The fish were fed ad libitum for 4 h per day using a conveyer belt feeder. Daily feeding and feed waste were recorded to estimate the feed conversion ratio (FCR), using a standard ATC correction factor. Fish were stripped for feces to calculate the apparent digestibility coefficient (ADC). The feed intake, weight gain, FCR, and specific growth rate (SGR) were calculated based on average fish weight and survival of the fish. Fish pigments were measured at the end of the feeding trial using Filet Minolta (*n* = 15) and NQCSalmonFan (*n* = 15). The fish were euthanized by benzocaine (Aquacen Benzocaine, Cenavisa, Tarragona, Spain) before dissection. Flesh samples from five fish per tank were collected for quantification of Ax and total fat in Norwegian Quality Cut (NQC). The color of the NQC flesh samples was also measured using the Roche color card (Hoffmann-La Roche, Switzerland).

### 2.2. Diet Preparation

Four diets were formulated for the feeding trial: a control diet which satisfy the nutritional requirements of rainbow trout, a 2% PS diet, a 2% CH diet, and a 2% DHA diet (Table 1). All diets were manufactured at BioMar Tech-Centre (Brande, Denmark). Feed near-infrared reflectance (NIR) and physical analysis were performed at Biomar BioLab. The composition of the four diets were analyzed at Eurofins AS (Vejen, Denmark). The PS diet contained slightly higher fat levels than the control and the CH diet, while the DHA diet contained slightly lower total fat content (Table 2). Additionally, the PS diet also had higher CH contents than control diet, and the DHA diet contained higher PS amount than the other diets. This is likely a technical issue as the machine may not have been properly decontaminated between each diet production.

### 2.3. RNA-Seq

Midgut tissue was sampled from 24 individual fish (two individual fish x 3 replicate tanks x 4 dietary groups) for RNA-seq analysis. The RNA extraction and library preparation were performed in the lab at Center of Integrative Genetics (CIGENE), Norwegian University of Life Sciences (Aas, Norway). Total RNA was extracted by using the RNeasy Universal Kits (Qiagen, Hilden, Germany), according to the manufacturer's instructions. The concentration of the extracted RNA was determined by using Nanodrop 8000 (Thermo Fisher Scientific, Waltham, USA). The integrity of RNA was determined by using Bioanalyzer 2100 (Agilent Technologies, Santa Clara, USA). All samples had RNA integrity (RIN) values higher than 8, which indicated high quality RNA for sequencing. Sequencing libraries were prepared by using TruSeq Stranded mRNA Library Prep Kit (Illumina, San Diego, USA). Library were sequenced using 100 bp single-end (SE) mRNA sequencing on Novaseq S1 full flow cell in Norwegian Sequencing Center (Oslo, Norway).

Raw sequences (fastq file) were processed using the bcbio-nextgen pipeline (https://github.com/bcbio/bcbio-nextgen). Raw reads were first aligned to the rainbow trout genome (USDA_OmykA_1.1) using STAR [19]. The resulting .bam files were subsequently used to generate raw gene counts using feature Counts (v1.4.4). Raw counts were further analyzed in R (version 1.2.5025) using edgeR package [20].

### 2.4. Proteomics

Intestine sample was added 75 μl 8% SDS and 100 mM Tris-HCl pH-7.5, and sonicated for 10 cycles (30 s ON/30 s OFF) using a Bioruptor sonicator. Protein precipitation was performed by using the methanol/chloroform precipitation method [21] on 30 μl of each sample. The precipitated protein pellet was further dissolved in 100 mM ammonium bicarbonate followed by reduction and alkylation using 10 mM dithiothreitol (DTT) (30 min, 55°C) and 20 mM iodoacetamide (IAA) (30 min, room temperature, dark room). After an overnight digestion, the sample was then acidified using acetic acid and desalted using in-house made StageTips [22].

After desalting, peptides were dried down in a SpeedVac centrifuge and resuspended in 0.1% formic acid. Concentration of peptides was measured using nanodrop A205. Peptides (1 μg) were analyzed on a LC-mass spectrometry (MS)/MS platform consisting of an Easy-nLC 1000 UHPLC system (Thermo Fisher Scientific, Waltham, MA, USA) interfaced with an QExactive orbitrap mass spectrometer (Thermo Fisher Scientific) and a nanospray electrospray ionization (ESI) ion source (Proxeon, Odense, Denmark). Peptides were injected into a C-18 trap column (Acclaim PepMap100, 75 μm x2 cm, C18, 3 μm, 100 Å, Thermo Fisher Scientific) and further separated on a C-18 analytical column (Acclaim PepMap100, 75 μm x50 cm, C18, 2 μm, 100 Å, Thermo Fisher Scientific) using a 120 min gradient from 5% buffer A (0.1% formic acid) to 50% buffer B (80% CH_3_CN, 0.1% formic acid). The flow rate was 250 nL per minute. The eluted peptides were analyzed on QExactive mass spectrometer operating in positive ion and data dependent acquisition mode using the following parameters: electrospray voltage 1.9 kV, higher-energy collisional dissociation (HCD) fragmentation with normalized collision energy 35, and automatic gain control target value of 3E6 for Orbitrap MS and 1E5 for MS/MS scans. Each MS scan (m/z 400–1600) was acquired at a resolution of 60,000 full width at half maximum (FWHM), followed by 15 MS/MS scans triggered for automatic gain control (AGC) targets above 2E3, at a maximum ion injection time of 50 ms for MS and 100 ms for MS/MS scans.

### 2.5. Statistics

The differential expression analysis (DEA) was performed using R (v.4.1.2) package edgeR [20]. Only genes with a minimum counts level of at least 1 count per million (CPM) in more than one-third of samples from each tissue were kept for further DEA. Genes with a false discovery rate (FDR), an adjusted *p* value (*q*) < 0.05 and absolute log2 fold change (|Log2FC|) > 1) were considered to be differentially expressed genes (DEGs) between test conditions.

Protein DAE was performed using R package differential expressed proteins (DEPs) [23]. Only proteins expressed in at least two-thirds of the samples from each dietary group were kept for further differential protein analysis. The data was first background corrected and normalized by variance stabilizing transformation [24]. Since strict cutoff of FDR-adjusted *p* value (*q*) < 0.05 gave too little DEP, we reduced the stringency of cutoff to *p* (uncorrected) < 0.005 to avoid being over conservative for the dietary contrasts.

All other statistical analyses were performed using R v.4.1.2. Datasets were first subjected to Shapiro–Wilk's test for normality. If data was normally distributed, the one-way analysis of variance (ANOVA) method was used to test the main effect, and Tukey's honestly significant difference (HSD) was then used for post hoc testing. If the data was not normally distributed, a robust Welch's ANOVA from R package WRS2 [25] was used for testing the main effect and Games–Howell method was used for post hoc testing.

## 3. Results

### 3.1. General Phenotypes of the Fish

In general, dietary inclusion of CH, PS, and DHA had little effect on fish growth and development. The fish under CH, PS, and DHA diets had similar SGR, thermal growth coefficient, whole-body protein, and whole-body fat compared to the fish fed the control diet (Table 3). However, dietary inclusion of CH, PS, and DHA significantly (*p* < 0.05) increased fecal fat levels of the fish especially those fed the PS diet with a fat content of 17% (Table 3). Higher fecal fat levels also contributed to higher FCR for the PS diet as compared to the control diet (0.86 versus 0.77, *p* < 0.05).

**Figure 1:**
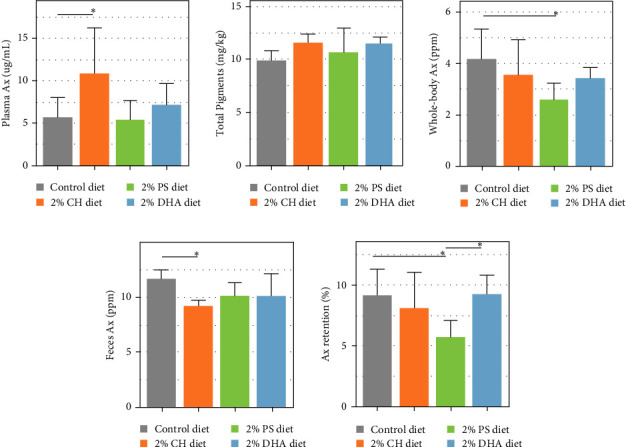
Effect of cholesterol (CH), phytosterol (PS), and docosahexaenoic acid (DHA) diets on astaxanthin (Ax) metabolism in rainbow trout. (A) Plasma Ax levels (ug/mL). (B) Roche color card score of the muscle salmon from Norwegian Quality Cut (NQC). (C) Whole-body Ax levels (ppm). (D) Ax levels in feces (ppm). (E) Ax retention in individual fish.  ^*∗*^*p* < 0.05.

Dietary inclusion of CH increased Ax absorption in rainbow trout. This was shown by elevated (*p* < 0.05) plasma levels of Ax compared to the fish fed control diet (Figure 1A). However, similar total pigments levels were observed between fish fed four diets (Figure 1B). A lower feces Ax content (*p* < 0.05) was also observed in fish fed CH (Figure 1C). It is however interesting to note that fish fed the control and CH diets had similar whole-body Ax contents and Ax retention values (Figure 1 D,E). On the other hand, feeding the PS diet did not affect plasma Ax levels in fish, but significantly reduced (*p* < 0.05) the whole-body Ax and Ax retention when compared to fish fed the control diet. Feeding DHA diets had no effect on content in fish. The color measurement using Minolta spectrophotometer did not identify significant differences between the color of head, back, and tail of fish fed four different diets (Figure S1).

### 3.2. Intestinal Transcriptome and Proteome of Fish Fed CH, PS, and DHA Diets

**Figure 2:**
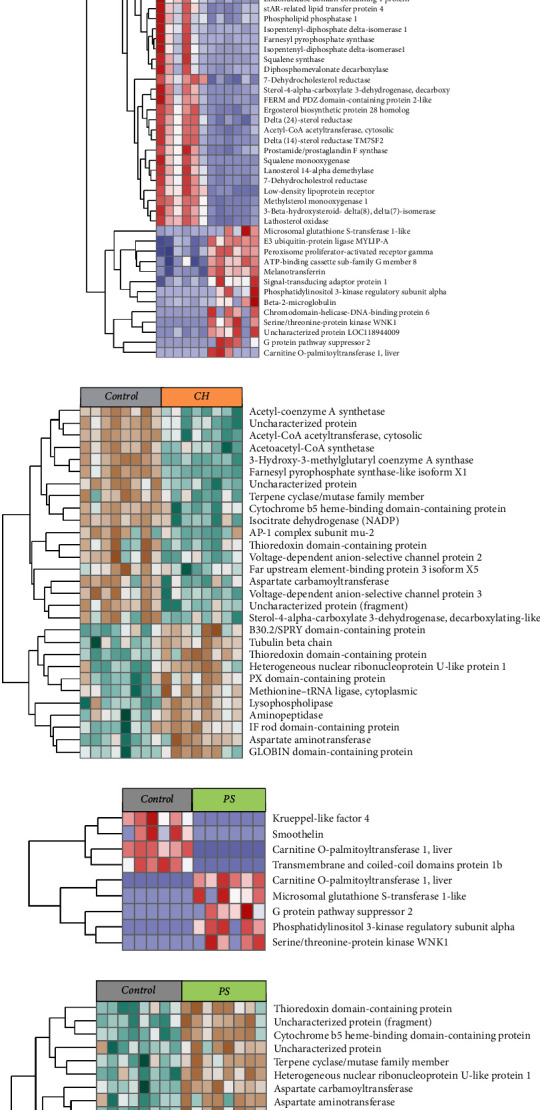
Effect of high cholesterol (CH), phytosterol (PS), and docosahexaenoic acid (DHA) diets on intestinal transcriptome and proteome. (A) Total number of differentially expressed genes (DEGs, *q* < 0.05) between fish fed CH, PS, or DHA diets as compared to the control diet. (B) Total number of differential expressed proteins (DEPs, *p* < 0.005) between fish fed CH, PS, or DHA diets as compared to the control diet. (C) Expression of DEGs in individual fish fed either control or CH diet. Data are row-scaled transcript per million (TPM) value. (D) Expression of DEPs in individual fish fed either control or CH diet. Data are row-scaled normalized protein expression value. (E) Expression of DEGs in individual fish fed either control or PS diet. Data are row-scaled TPM value. (F) Expression of DEPs in individual fish fed either control or PS diet. Data are row-scaled normalized protein expression value. (G) Expression of DEGs in individual fish fed either control or DHA diet. Data are row-scaled TPM value. (H) Expression of DEPs in individual fish fed either control or DHA diet. Data are row-scaled normalized protein expression value.

A total of 63 DEGs (*q* < 0.05) were identified between the intestines of fish fed the CH diet and the control diet (Figure 2A). Thirteen genes were upregulated in fish fed the CH diet, while 50 genes were downregulated. Only nine DEGs were identified between fish fed the PS diet and the control diet, which included five upregulated and four downregulated genes (Figure 2A). Feeding the DHA diet to rainbow trout resulted in nine DEG compared to fish fed the control diet, which included two upregulated and seven downregulated genes (Figure 2A).

Under the same stringency cutoff (*q* < 0.05), only 7, 1 and 0 DEPs were identified between fish fed CH, PS, and DHA diet, respectively, compared to fish fed the control diet. To avoid being over conservative with the dietary contrasts, the stringency of cutoff was reduced to *p* (uncorrected) < 0.005, which identified 29, 17, and 6 DEPs in fish fed the CH, PS, and DHA diets, respectively, compared to fish fed the control diet (Figure 2B).

Feeding the CH diet had a more pronounced impact on fish gut transcriptome and proteome than feeding the PS and DHA diets. Further analysis of the DEGs between fish fed the CH diet compared to fish fed the control diet revealed that 24 DEG (37%, Figure 2C) and six DEP (21%, Figure 2D) were directly involved in the de novo CH synthesis pathway. These genes were consistently downregulated in fish fed the CH diet, which suggested a substantial inhibition of CH biosynthesis in response to increased dietary intake of CH. Two low-density lipoprotein receptor (*ldlr*) genes which involved in low-density lipoprotein (LDL) regulation [26] were downregulated when fed the CH diet as compared to fish fed the control diet (Figure 2C). The *fads2* gene which plays a crucial role in highly unsaturated fatty acid (HUFA) synthesis was also reduced when fish were fed the CH diet [27]. The upregulated DEGs were enriched in fatty acids degradation pathways (Figure 2C). This included the *cpt1* gene which encodes the carnitine palmitoyltransferase 1, an rate-limiting enzyme in the fatty acid *β*-oxidation pathway [28]. A key transcription factor which involves in lipid metabolism, *pparg* was also upregulated in fish fed the CH diet. Additionally, gene *abcg8* which is responsible for the efflux of CH back into the intestinal lumen, was upregulated in fish fed the CH diet [29] (Figure 2C). However, the proteomic sequencing did not identify Ldlr, Abcg5, Abcg8, or Ppar proteins in intestine of the fish fed the CH diet as compared to fish fed the control diet (Figure 2D).

Most of the DEG identified between fish fed the PS diet and control diet were associated with inflammatory or fatty acid metabolism pathways (Figure 2E). The *klf4* gene, which is a key transcription factor for anti-inflammatory processes, was downregulated in fish fed the PS diet [30] (Figure 2E). Two isoforms of *cpt1* genes were found in both upregulated and downregulated gene list, suggesting an altered fatty acid *β*-oxidation pathway. Other four upregulated DEGs microsomal glutathione S-transferase 1 (*mgst1*), g protein pathway suppressor 2 (*gps2*), WNK lysine deficient protein kinase 1 (*wnk1*), and phosphoinositide-3-kinase regulatory subunit 1 (*pik3r1*) are all known to play roles in inflammatory processes [31–34] (Figure 2E). None of the genes in CH synthesis pathway was differentially expressed in fish fed the PS diet as compared to fish fed the control diet. However, the DEP 3-hydroxy-3-methylglutaryl coenzyme A synthase (Hmgcra) which was a rate-limiting enzyme involved in CH biosynthesis, was identified to be downregulated in fish fed the PS diet (Figure 2F). On the other hand, protein acetyl coenzyme A synthetase (Acat), which involved in the first step of CH biosynthesis was upregulated in fish fed the PS diet (Figure 2F).

None of the DEGs between fish fed the DHA and the control diets were directly involved in lipid metabolism regulation (Figure 2G). The DEGs, *gimap8*, *gimap7*, *gal9b*, *gps2*, *wnk1*, and dedicator of cytokinesis 2 (*dock2*), were all associated with immune responses (Figure 2G). However, the DEP Hmgcra protein was significantly upregulated in fish fed DHA diet (Figure 2H).

## 4. Discussion

Three different diets CH, PS, and DHA were tested in the present study on the absorption and retention of Ax in rainbow trout. Dietary inclusion of CH clearly improved the plasma Ax levels. Such beneficial effects by dietary CH were also observed Atlantic salmon [35] and Giant tiger prawn [36]. Similar to CH and carotenoids, Ax is transported in different lipoproteins in fish plasma [37]. One hypothesis for the mechanism underlying the beneficial effect of dietary CH was that it increased plasma Ax by promoting the production of lipoproteins [35]. This was supported by the present study since the transcriptomic analysis has identified downregulation of *ldlr* gene in intestine of the fish fed the CH diet as compared to the control diet. Since LDLR is known to be important for lipoprotein cleavage, the decreased *ldlr* expression could increase the lipoprotein half-life thereby the Ax retention in fish. The hypothesis was supported by previous zebrafish and mice study that knockout of the *ldlr* gene significantly increased the plasma CH and lipoprotein levels ([38, 39]. A previous mice study has also shown that intestinal-specific overexpression of *ldlr* gene caused decrease of plasma LDLs and CH [26]. The change of *ldlr* expression was likely due to sterol regulatory element binding protein (Srebp) regulation [40], which is known to be regulated by cellular CH levels [41]. Both *srebp* and *insig* genes involved in SREBP expression [42], were significantly downregulated (*q* < 0.05) in fish fed the CH diet compared to fish fed the control diet. Previous studies have shown that genetic variation in BCO1, the primary enzyme responsible for carotenoid degradation in the intestine, plays a critical role in regulating Ax metabolism and flesh color in salmonids [13]. However, neither *bco1* nor *bco1-like* genes were changed in fish fed the CH, indicating that dietary CH had no effect on Ax degradation pathway in the intestine.

Although the present study identified higher Ax levels in plasma of fish under the CH diet, there was no differences in flesh color of the fish (Figure 1). This suggests that there are other factors which limit the uptake and retention of Ax in muscle. Although gene or protein levels in muscle was not measured in the present study, it could be speculated that the presence of a membrane transporter protein, possibly Ldlr, or Sr-b1 [8, 9], was also inhibited by dietary CH and caused the decreased uptake of Ax from plasma into muscle. Another possible explanation is that plasma CH could compete with Ax for the entry into muscle cells and reduce the uptake of Ax in the muscle.

Fish fed the PS diet had decreased whole-body Ax level and Ax retention (Table 3). This could be due to a slight inconsistency in Ax levels between the control diet (59.1 ppm) and the PS diet (52.9 ppm). Another possible mechanism is that dietary PS are known agonists for the liver X receptor (LXR) which activates several sterol transporters including ABCG5, ABCG8, ABCG2, and ABCG1 [43, 44]. The activation of ABCG2 could cause higher Ax excretion back to intestinal lumen, thereby, decreasing Ax retention [16].

Surprisingly, fish under the DHA diet had similar level of whole body and plasma Ax levels, despite the DHA diet had lower Ax (47 ppm) compared to the control diet (59.1 ppm). This suggests a potential beneficial effect of dietary DHA on Ax absorption or retention, though the mechanism remains unclear. Dietary organic acids have been known to stimulate innate immune response, while DHA is known for its strong anti-inflammatory effect [45–47]. This was also demonstrated in the present study, where the anti-inflammatory gene *gps2* [33, 48] was significantly up-regulated in fish fed the DHA diet, while pro-inflammatory gene *dock2* which responsible for G protein activation [49] was significantly downregulated. A previous study in mouse and human macrophages suggested that GPS2 induced Abca1 protein by modulating the NF-*κ*B pathway [33]. As Abca1 is suggested to be involved in the HDL pathway for intestinal absorption of xanthophylls [50, 51], the beneficial effect of dietary DHA could be explained by the activation of Gps2/Abca1 pathway which facilitate the Ax absorption in intestine.

Taken together the results of three diets in this study, it could be concluded that the beneficial or inhibitory effects of different diets on Ax retention is by modulating the membrane proteins in intestine such as ABC transporters to increase or decrease the Ax absorption or excretion. The ABC transporters may have different substrate specificity; however, structurally similar compounds such as PS, CH, and Ax could share the same transporting tunnels or regulate the transporter through same signaling process. For example, both PS and CH are known to increase ABCG5 and ABCG8 expression (Figure 2) [43, 44] and ABCA1 is able to transport both xanthophylls and CH through basolateral membrane of the intestine [50, 51]. From the study we found that dietary CH clearly promote Ax absorption by increasing plasma Ax in rainbow trout. This is suggested to be caused by the inhibition of *ldlr* gene which decreased the excretion of Ax-containing lipoproteins from intestine, thereby, increasing the amount of circulating Ax in plasma. However, feeding CH did not cause an increase of Ax content or redness of the muscle flesh, which suggested another factor, possibly a membrane protein that limited the transport of Ax from plasma to muscle tissue.

## 5. Conclusion

In conclusion, this study has suggested that CH promotes Ax absorption by regulating lipoprotein retention and provide evidence for improving Ax absorption via dietary modulation. Dietary PS likely limited the Ax absorption, while dietary DHA may have beneficial effect on Ax absorption. However, further studies are needed to understand the molecular mechanism underlying the inhibition or beneficial effect. It is, therefore, concluded that the absorption, transport, and retention of dietary Ax could be affected by other nutrients, especially lipids in the diet through various metabolic mechanisms.

## Figures and Tables

**Table 1:** Formula of the control, cholesterol (CH), phytosterol (PS), and DHA diets (%).

Ingredients	Control	CH	PS	DHA
Fish meal	15	15	15	15
Krill meal	2	2	2	2
Vegetable protein^a^	42.86	41.83	41.83	41.83
Wheat milling quality	8.5	8.5	8.5	8.5
Fish Oil	8.36	8.37	8.37	8.37
Rapeseed oil, crude	19.49	17.67	17.67	17.67
Vitamins, minerals, premix, and amino acid	2.7	3.5	3.5	3.5
MSP	1.02	1.04	1.04	1.04
Yttrium	0.05	0.05	0.05	0.05
Lucantin pink CWD 10%	0.05	0.05	0.05	0.05
CH (Deichman)	0	2	0	0
PS (Nanjing NutriHerb BioTech)	0	0	2	0
DHA (OmegaVie, Polaris)	0	0	0	2

Abbreviations: CH, cholesterol; DHA, docosahexaenoic acid; EPA, eicosapentaenoic acid; MSP, monosodium phosphate; PS, phytosterol.

^a^Vegetable protein includes protein concentrates of soya, sunflower, maize, peas, and guar. Wheat milling and dehulled horse beans are used as binders.

**Table 2:** Composition of the control, cholesterol (CH), phytosterol (PS), and DHA diets.

Type of measurement	Control	CH	PS	DHA
Moisture (% of diet)	6.9	5.8	6.1	6.3
Protein (% of diet)	40.7	41.2	41.8	43.6
Fat (% of diet)	30.6	30.4	33.1	28.8
Ash (% of diet)	5.1	5.1	5.0	5.1
CH (mg/100 g)	93	1700	670	260
PS (mg/100 g)	197	219	1511	605
EPA (% total fatty acids)	3.77	3.93	3.94	3.45
DHA (% total fatty acids)	3.57	3.76	3.71	6.38
Ax (ppm)	59.1	52.4	52.9	47.8

Abbreviations: Ax, astaxanthin; CH, cholesterol; DHA, docosahexaenoic acid; EPA, eicosapentaenoic acid; PS, phytosterol.

**Table 3:** Phenotypes of the fish fed the control, cholesterol (CH), phytosterol (PS), and DHA diets.

Phenotypes	Control	CH	PS	DHA
Thermal growth coefficient	2.7 ± 0.4^a,b^	2.9 ± 0.1^a^	2.2 ± 0.3^b^	2.4 ± 0.6^a,b^
FCR	0.77 ± 0.04^a^	0.83 ± 0.02^a,b^	0.86 ± 0.06^b^	0.79 ± 0.07^a,b^
SGR (%/day)	1.43 ± 0.18^a,b^	1.51 ± 0.05^a^	1.21 ± 0.12^b^	1.30 ± 0.29^a,b^
SFI (%/day)	1.10 ± 0.14^a,b^	1.26 ± 0.06^a^	1.03 ± 0.03^b^	1.02 ± 0.18^a,b^
Fat (%)	12.9 ± 0.6	14.0 ± 0.7	13.1 ± 1.9	13.1 ± 0.9
Whole-body protein (%)	45.6 ± 2.6	44.2 ± 3.7	47.1 ± 1.7	46.0 ± 5.3
Whole-body fat (%)	49.3 ± 2.1	52.1 ± 4.4	47.4 ± 1.9	50.5 ± 6.6
Feces protein (%)	17.2 ± 0.5^a^	16.1 ± 0.5^b^	16.0 ± 1.1^b^	16.5 ± 0.4^a,b^
Feces fat (%)	10.3 ± 1.1^a^	14.6 ± 0.8^b^	17.0 ± 0.5^c^	12.1 ± 0.6^d^
Feces ash (%)	22.5 ± 0.4^a^	21.1 ± 0.1^b^	21.0 ± 0.2^b^	23.0 ± 0.8^a^
Fat ADC (%)	90.5 ± 4.8	89.1 ± 4.5	83.7 ± 6.6	87.0 ± 6.5
Protein ADC (%)	88.5 ± 5.4	91.1 ± 4.0	87.9 ± 5.0	88.2 ± 5.9

*Note*: All parameters were measured in the proportion of dry matter. Different alphabets indicate statistical significance (*p* < 0.05) between groups.

Abbreviations: ADC, apparent digestibility coefficient; FCR, feed conversion ratio; SFI, specific feed intake; SGR, specific growth rate.

## Data Availability

All the raw data and codes for transcriptomics and proteomics analysis are available at https://github.com/jinyangye119/Rainbowtrout_asta.

## Supporting Information

Supporting Information Figure S1. Flesh color (Minolta) of the fish fed four different diets (*n* = 45 fish each dietary group).
