# Supplementary material for: Effect of Dietary Cholesterol, Phytosterol, and Docosahexaenoic Acid on Astaxanthin Absorption and Retention in Rainbow Trout
Source: Aquac Nutr. 2024 Oct 15;2024:8265746. doi: 10.1155/2024/8265746 (PMC11496587; doi:10.1155/2024/8265746)
Supplement: Supporting Information — Figure S1. Flesh color (Minolta) of the fish fed four different diets (n = 45 fish each dietary group). [file 8265746.f1.docx]

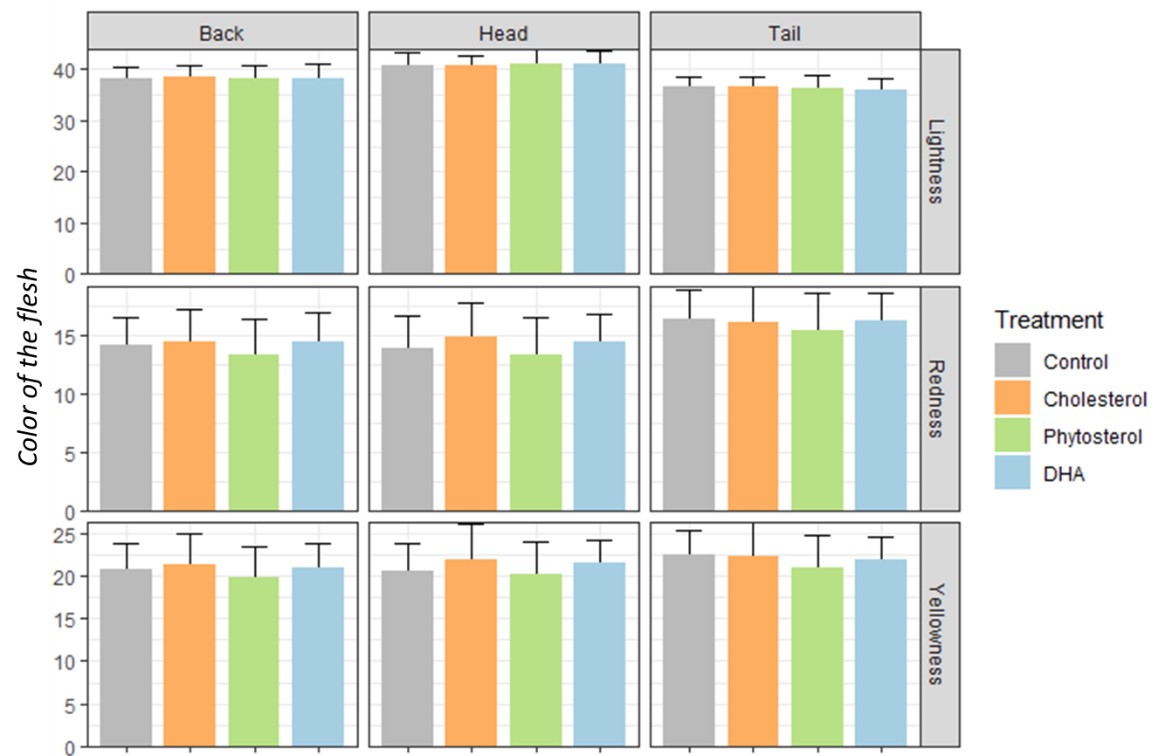


Supplementary Figure 1. Flesh color (Minolta) of the fish fed 4 different diets (n= 45 fish each dietary group).
